# Demographic rise of sea urchin *Centrostephanus sylviae* on Robinson Crusoe and Santa Clara Islands at the Juan Fernandez Archipelago: A biophysical and ecological approach

**DOI:** 10.1371/journal.pone.0325556

**Published:** 2025-06-25

**Authors:** Valentina Nuñez-Espinosa, Carolina Parada, Braulio Tapia, Billy Ernst, Javier Porobic, David Véliz, Iván Hinojosa, Leonardo Yévenes-Vega

**Affiliations:** 1 Programa de Magister en Ciencias mención en oceanografía, Departamento de Oceanografía, Universidad de Concepción, Concepción, Chile; 2 Departamento de Geofísica, Universidad de Concepción (UdeC), Concepción, Chile; 3 Center for Ecology and Sustainable Management of Oceanic Islands (ESMOI), Departamento de Biología Marina, Facultad de Ciencias del Mar, Universidad Católica del Norte, Coquimbo, Chile; 4 Climate action planning Program (CLAP), ANID-CENTROS REGIONALES R20F0008, Coquimbo, Chile; 5 Departamento de Oceanografía, Universidad de Concepción (UdeC), Concepción, Chile; 6 Commonwealth Scientific and Industrial Research Organization (CSIRO) Environment, Hobart, Australia; 7 Centre for Marine Socioecology, University of Tasmania, Hobart, Australia; 8 Departamento de Ciencias Ecológicas, Facultad de Ciencias, Universidad de Chile, Santiago, Chile; 9 Departamento de Ecología, Facultad de Ciencias, Universidad Católica de la Santísima Concepción, Concepción, Chile; 10 Centro de Investigación en Biodiversidad y Ambientes Sustentables (CIBAS), Universidad Católica de la Santísima Concepción, Concepción, Chile; 11 Centro de Investigación Oceanográfica COPAS COASTAL, Universidad de Concepción, Concepción, Chile; University of Minnesota, UNITED STATES OF AMERICA

## Abstract

The large increase in the population of long-spined sea urchins (*Centrostephanus sylviae*) has gained significant attention in the past decade due to the rise in the number of individuals reported as bycatch in Juan Fernandez rock lobster traps (*Jasus frontalis*) and the risks associated with changes in the ecosystem structure due to the increase in bleaching of reefs in the Juan Fernandez Archipelago (JFA). We explored the demographic surge of the *C. sylviae* population on Robinson Crusoe and Santa Clara islands (RC-SC) through changes in the relative abundance of adult sea urchins during the years 2015–2022. To seek an explanation for this phenomenon, we explored the potential contributions of early life stages of sea urchins to the adult population via biophysical modeling. We performed simulations of larval dispersal patterns and connectivity between release and recruitment zones for three study years (2013, 2015, and 2018). The results from larval drift simulations combined with observation data from the crustacean fishery monitoring program helped identify recruitment zones (primarily located in the eastern, southeastern, and southwestern areas of RC-SC). Also, we explored the relationship between the relative abundance of adult sea urchins and traits associated with lobsters due to the predator-prey relationship evident in other ecosystems (i.e., in Tasmania and New Zealand). We explored the potential control by lobsters of the sea urchin population size through generalized linear models by analyzing several predictor variables. The results showed that once all zones were combined there was an inverse correlation between the relative abundance of sea urchins and the largest lobsters found in the traps around the islands. This work highlights the complex ecological dynamics resulting from the increase in the *C. sylviae* population in the JFA system, emphasizing the importance of addressing this issue through ecosystemic and socio-ecologically integrated approaches.

## Introduction

**Fig 1:**
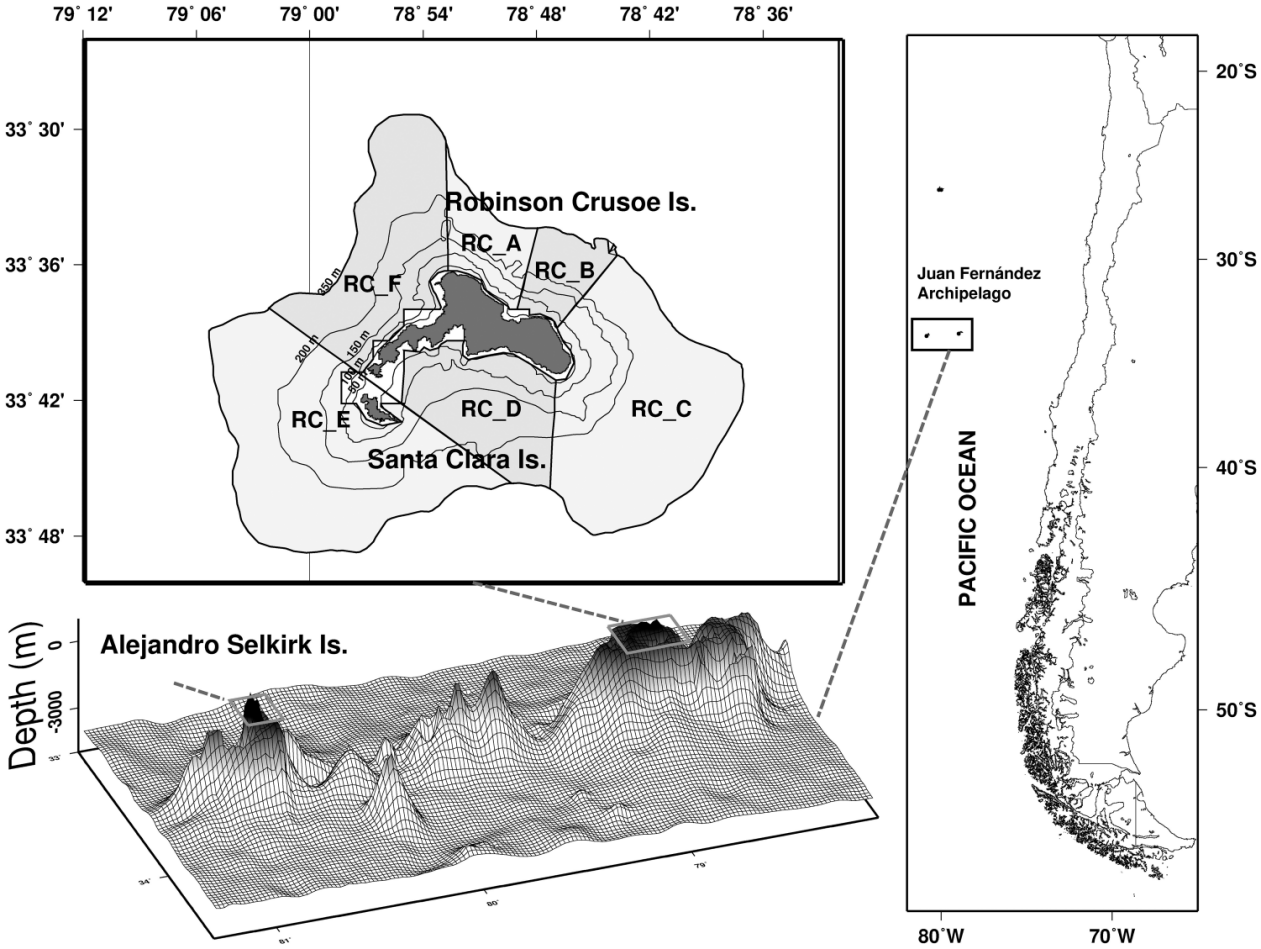
Geographic location of Juan Fernández (JFA) Archipelago on the JF ridge. The upper left panel corresponds to the study area (Robinson Crusoe and Santa Clara Islands) and 6 zones defined for prediction analysis. Figure generated under a CC BY license, with permission from Billy Ernst, original copyright 2024.

The Chile-Peru current system (CPCS) forms part of the eastern boundary upwelling ecosystem [1] characterized by oceanographic processes such as mesoscale eddies, filaments, and meanders that propagate from the South American coast with a high level of kinetic energy (50−110 cm2 s-2) [2] and interact with oceanic islands. The Juan Fernandez Archipelago (JFA) comprises the Robinson Crusoe and Santa Clara islands (RC-SC) located about 700 km off the coast of Central Chile, and the Alejandro Selkirk Island (AS) situated 162 km to the west (Fig 1). The JFA ecoregion is considered a biodiversity hotspot due to its remote location and high level of endemism [1,3,4]. UNESCO declared the JFA as a biosphere reserve in 1977 [5] and in 2016, the Mar de Juan Fernandez Marine Park was created with a 2.4-million-hectare surface area.

The lobster *Jasus frontalis* is among the most iconic endemic species within the JFA and represents a keystone species for the artisanal fishing sector, comprising much of the crustacean fishery of the archipelago [6]. The distribution of *J. frontalis* occurs from the coastal edge around the islands, up to 200 m in depth [7]. The lobster is distributed on hard ocean floor habitats such as rocky reefs, with seaweeds and associated fauna [8]. Another endemic species of this archipelago is the long-spined urchin *Centrostephanus sylviae*, a species that has gained relevance during the last decade due to the sustained increase in the capture rates of specimens found as bycatch in the lobster traps and the increase of overgrazed and depleted bottoms along the coasts of the islands [9].

Ernst et al. [10] indicated that the increase in the sea urchin population has become relevant among the different stakeholders of the fishery and the Juan Fernandez community. The increase in the presence of *C. sylviae* adults in lobster traps around RC-SC has been substantial, constituting up to 85% of the bycatch and generating significant changes in the rocky subtidal landscape and negatively affecting tourism. Ecosystem modeling of *J. frontalis* at the Juan Fernández ridge suggests that the high exploitation rate to which lobster of the commercial size are harvested (≥ 115 mm cephalothoracic length, CL) would have reduced the population control of the urchin, facilitating the population growth of this species [11]. Thus, adult lobsters are potentially crucial factors in controlling the sea urchin increase [12]. In other similar Indo-Pacific ecosystems (i.e., Tasmania; [13]), the control of lobsters over sea urchins decreased the effect of overgrazing on macroalgae, and with it, the reduction of the chances of bleaching the rocky substrate, therefore avoiding significant changes in the structure of the ecosystem [12]. Global evidence suggests that, in general, underwater macroalgae forests are essential for the recruitment, settlement, and survival of the early stages of crustaceans, since they provide food, shelter, and recruitment sites [14]. Nonetheless, the effect that the increase in overgrazed bottoms may have on the recruitment of *J. frontalis* and the possible increase in urchin larvae in the water column is yet unclear.

The first record of long-spined urchins in these Chilean islands was described by Fell (1975) [15], indicating their presence on San Felix Island and the JFA. Recent studies indicate that even though both island systems are 819 km apart, the populations of *C. sylviae* are highly connected, given the apparent high level of gene flow. The extensive larval period of the species and the meso- and submesoscale structures facilitate larval transport to form a single genetic population [16]. For the genus *Centrostephanus*, the distribution has been described around shallow-rocky bottoms with the presence of kelp and coralline algae [17]. The reproductive period for this genus starts at the beginning of the southern winter, with a peak of spawning at the beginning of June. A meroplanktonic larva is formed, remaining in the water column for approximately four months [18–20]. The highest gonadosomatic index of *C. sylviae* occurs from February to May, decreasing during the southern winter (June, July, and August) [9]. However, there is no more available information regarding the life history and ecology of the species in the area. It is thought that physical processes could modulate the transport of the reproductive products of *C. sylviae* [11,16,21–23]. Thus, the dispersion and retention capacity of *C. sylviae* at the larval stage would determine the population distribution of adult sea urchins and hence may show that their spatial niche overlaps with that of adults of *J. frontalis*.

This research explored the increase in the relative abundance of *C. sylviae* around the islands RC-SC during the past decade (2015–2022) and their relationship with the dispersion/connectivity and larval retention within the islands using simulation modeling. In addition, the present work examined the possible influence of several variables on the sea urchin population size, including the size of adult lobsters, by utilizing generalized linear models. The findings of this study will provide valuable insights into the increase in degraded bottoms of rocky substrates and the potential impact on the ecosystem of the archipelago. Stakeholders can use this information to develop effective management and conservation strategies.

## Materials and methods

### Data based on the monitoring program

For the statistical analysis and setup of the biological and ecological components of the model, we used the database recorded by the fisheries biological monitoring program for the JFA ecosystem from 2015 to 2022 for the island subsystem comprising Robinson Crusoe and Santa Clara (RC-SC) [9,10]. From this database, information was obtained per lobster trap haul, including cephalothoracic length (CL) of retained (≥115 mm CL) and non-retained lobsters (<115 mm CL) and sea urchin (*C. sylviae*) bycatch (numbers) in addition to geographic information concerning the areas and depths in which the traps were set.

### Hydrodynamic model

The dynamic representation of the circulation of the study area (JFA) was obtained from the products of two global reanalyses (i.e., CGLOPHY30 and CGLOPHY024) distributed by Copernicus Marine Environment Monitoring Service (CMEMS; http://marine.copernicus.eu/). CGLOPHY030 corresponds to a daily global reanalysis model from which data were used for simulation experiments for 2013, 2015, and 2018 [24]. GLOPHY024 is a daily global forecast model that has a regular medium resolution grid of 1/12° (~8 km) and 50 levels of depth (0–5500 m) [25]. The products of both models of the reanalysis were based on the hydrodynamic model Nucleus for European Modelling of the Ocean (NEMO) version 3.1 that assimilates altimetry, satellite data of sea surface temperature, and the salinity of the water column [26].

### Biophysical model, parameters, and index

An individual-based model (IBM) coupled to the reanalysis products was implemented using the Ichthyop simulation program that allows for the development of spatiotemporal simulation experiments forced by the outputs of the hydrodynamic model, i.e., fields of speed, temperature, and salinity, among others [27]. We assessed the effects of physical processes on the larval-planktonic stage of *C. sylviae* from the RC-SC insular platform. For the configuration of the IBM, we defined the following biological characteristics of the larval stage of *C. sylviae*: 1) the release date; 2) the release area; 3) the number of larvae released; 4) the place of recruitment. Recruitment was identified from two calculated indices of the statistical values from the IBM model. The first corresponds to the index of connectivity within and between the study areas and is given by the number of larvae released that arrived in an area different from their release. The second consists of the retention index calculated from the number of larvae present in the same area in which they were released. The indices were estimated considering 115 days (~4 months) with a window of five days prior and five days after the end of the simulation considering that larvae generate pods within ~100 days and the recruitment takes approximately five days [20].

### Simulation experiments and assessed parameters

We set up simulation experiments to evaluate connectivity and retention in RC-SC using the years 2013, 2015, and 2018 as case studies. We chose the year 2013 because variation in the time series’ abundance was found in cycles lasting two to three years. The year 2015 was selected for being the first year of the time series of the biological monitoring program. Finally, 2018 was the first year with a significant increase in the relative abundance of sea urchins in the time series. The areas of release were defined based on the information recorded by the biological monitoring program of the fisheries and the ecosystem associated with the JFA [10]. Six zones were established around RC-SC, and these were used to identify the recruitment of the propagules (Fig 1).

To assess the connectivity between the areas around RC-SC, 4-month larval period was considered. Each IBM simulation lasted for 122 days (~4-month larval period), following the duration reported for *Centrostephanus rodgersii* [20]. In the absence of specific information on *C. sylviae*, data from the congeneric species *C. rodgersii* was used as a reference due to the similarity in their life history traits. The simulations were conducted with a time step of 120 seconds. The model outputs being recorded every six hours. A daily release of 6000 particles was arranged for 92 consecutive days, within a depth range of 0–80 m. The particles were released throughout the entire study area, covering all previously defined release zones during the spawning peaks in June, July, and August. A total of 552000 particles were released during the simulation period for each case study. [18]. For the IBM developed in this study, a Lagrangian model was used to simulate the transport of passive larvae, with no vertical migration or active swimming. In addition, larval mortality rate functions associated with temperature were not included.

### Movement equations

The resolution of the position of particles in each time step used in the model was a function of the orthogonal east-west (*u*) and north south (*v*) velocity components in addition to the vertical component (*w*) from the hydrodynamic model output:


$$\begin{array}{c} \frac{dx}{dt}=u,\;\frac{dy}{dt}=v\;,\frac{dz}{dt}=w \end{array}$$ (1)

The integration of the position of the particles in each time step (*∂t*), was carried out with a fourth order Runge Kutta approximation [28], where y represents the dependent variable, *n* corresponds to the index of the discrete-time passage and *k* alludes to the coefficients that represent the approximations of the slope of the function at the different points throughout the time interval:


$$y^{n+1}=y^{n}+(1/6)(k^{1}+2k^{2}+2k^{3}+k^{4})$$ (2)

### The relative abundance of sea urchins and factors that affect their population dynamics

The relative abundance of sea urchins was estimated based on the frequency of sea urchin catches obtained from lobster trap bycatch. A generalized linear model was implemented (GLM; [29]) to determine the factors affecting the bycatch rates of long-spined urchins associated with the eventual control that large lobsters may have on the sea urchin relative abundance. Therefore, five predictor variables were considered, year (*aj*, 2015–2022), month (*mk*, October-May), zone (*zl*, RC_A, RC_B, RC_C, RC_D, RC_E and RC_F), stratum of bathymetric depth (dm, 0–50; 50–100; 100–150; and 150–200 m), and two lobster size ranges (*sn*, small, ≤ 107.5 mm LC and large, > 107.5 mm LC). The number of urchins per trap was set to a Poisson random variable (counts), with values equal to or greater than zero (>0). However, many trap hauls had zero urchin bycatch; therefore, the relative abundance of urchins was modeled using different likelihood options: Poisson, zero-inflated Poisson, negative binomial, and zero-inflated negative binomial. Through the Akaike information criterion (AIC; [30]) and overdispersion analysis, the GLMs with zero-inflated binomial probability distributions were determined to be the most appropriate. Thus, models with negative binomial distributions allow the relation of the response variable (Y) with the explanatory variables X using a logarithmic link function, where βi are the coefficients of the function estimated from the five predictors {*aj*, *mk*, *zl*, *dm*, *sn*}, while 𝜖 corresponds to the distribution of the error of the dependent variable belonging to the family of exponential distributions:


log(y)=Xβi+∈ (3)

The zero-inflated negative binomial distribution has parameters (the mean) and (the scaled variance) and another of form *ϕ* that represents the dispersion of the error incorporated into the variance. In this case, the distribution was truncated to zero (y = 0), meaning that the probability that urchins will not be observed in lobster traps is given by:


Y~nbinom (λ,ρ,ϕ) (4)

The parameters of the linear models were estimated using the glmmTMB function in the R statistical programming environment ([31]; R Development Core Team, 2023). This function allows a wide variety of available probability distribution families, including zero-inflated forms. After the model parameterization, comparisons were made through the AIC using the model’s diagnostic values for validation.

## Results

### Characterization of relative abundance of sea urchin *Centrostephanus sylviae*

**Fig 2:**
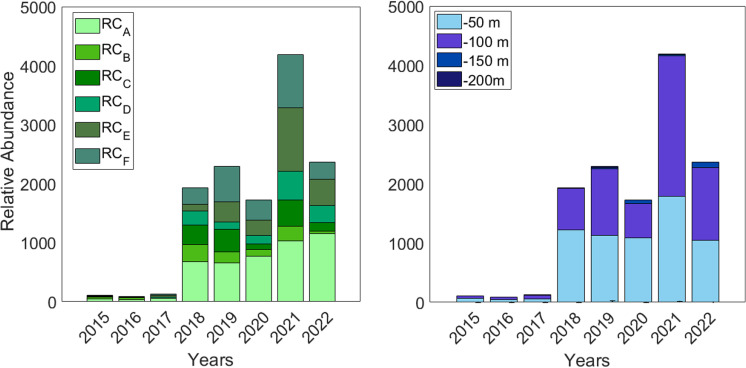
Relative abundance of sea urchin *Centrostephanus sylviae* captured on lobsters’ traps during the 2015-2022 period. A) Relative abundance of *C. sylviae* by year for each zone around RC-SC, B) Relative abundance of *C. sylviae* by depth strata for each zone around RC-SC.

The time series of relative abundance of sea urchins from the lobster traps spanned from 2015 to 2022. During the first three years of this time series (2015, 2016, and 2017), no significant increase in the proportion of *C. sylviae* specimens was observed. Starting in 2018, a significant increase in relative abundance (~ 2000 individuals) was recorded, with RC_A emerging as the zone with the highest presence of captured individuals. This trend persisted throughout the time series. Furthermore, unlike the previous peak in 2018, zone RC_E and RC_F also emerged as key areas with substantial presence of individuals (Fig 2A).

Regarding depth, a consistent trend was observed throughout the study period in terms of the strata at which most individuals were found (0–100 m). Following the peak in relative abundance identified in 2018, the majority of individuals were located in the first depth strata (0–50 m) until 2021, when a shift in the predominant depth of occurrence was observed (50–100 m), their quantities were marginal (Fig 2B).

### Predictions of the relative abundance of sea urchin adults

To determine whether elements of the RC-SC system might be influencing the relative abundance of urchins collected as bycatch in the lobster fishery, four predictor variables (year (*aj*), month (*mk*), depth (*dm*), and lobster size (*sn*)) were examined independently. Both simple and multiple linear regression analyses were used to analyze the predictor variables.

The simple linear regression models showed that the temporal factor (*aj*) had a significant influence on the abundance of *C. sylviae* in RC-SC for each of the study zones independent of the others since they were the models with the best-fit value. Once the six zones around RC-SC were integrated and considered as a factor, the year variable remained the parameter that had the greatest influence on the variability of urchin abundance over the given time series (2015–2022) (S1 Table).

The multiple regression models revealed that the combination of predictors varied among the zones; nevertheless, the AIC value indicated that model M5 that included all relevant predictor variables and considered the six zones surrounding RC-SC as a single factor was the best model (Table 1). To quantify the relationship between sea urchin abundance and the predictor variables, we used the values of the estimated coefficients of each level for each variable. Consistent with the simple linear regression analyses, the temporal factor (*aj*) had the most impact on urchin abundance, particularly the year 2021 (3.439955), followed by the year 2022 (3.124251). The lobster size (*sn*) was also a significant component; more specifically small size (0.167113). Despite its low coefficient value, helped to explain the fluctuations in *C. sylviae* abundance around RC-SC (S2 Table).

**Table 1:** Relative abundance of sea urchin GLM results of the best model fit. Results of models fit a relative abundance response variable (sea urchin) using simple (*m*) and multiple (*M*) regression models by zone, where the covariables are year factor (*a*_*j*_), month (*m*_*k*_) depth (*d*_*m*_) and rock lobster size (*s*_*n*_). AIC corresponds to Akaike information criteria and df to degrees of freedom. Bold and italic indicate the best model fit for each level.

Simple regression					Multiple regression			
Zone	Model	Covariate	df	AIC	Model	Covariate	df	AIC
All zones	**m1**	**~ a**_**j**_	**10**	**12949**	M1	~**a**_**j**_	10	12949
	m2	~ m_k_	10	13790	M2	~**a**_**j**_ + m_k_	17	12825
	m3	~ *z*_*l*_	8	13699	M3	~**a**_**j**_ + m_k_ + d_m_	22	12614
	m4	~ d_m_	6	13491	M4	**~a**_**j**_ + m_k_ + z_l _**+ **d_m_	25	12429
	m5	~ s_n_	4	13908	**M5**	**~a**_**j**_ ** + m**_**k**_ ** + z**_**l **_ **+ d**_**m**_ ** + s**_**n**_	**26**	**12425**

### Simulation of sea urchin larval transport, connectivity, and density

A significant degree of advection around the RC-SC islands was observed in the larval simulation experiments carried out for the three years (2013, 2015, and 2018) (>90% in each case). Transport via anticyclonic circulation was demonstrated by the simulated larval tracks over the islands. Furthermore, both in the retention region and the offshore area, larval dispersal displayed interannual variation. The year 2018 was the year with the most retention of the three (4.43%), with 2015 being the second highest (2.79%) and 2013 the least (0.38%).

**Fig 3:**
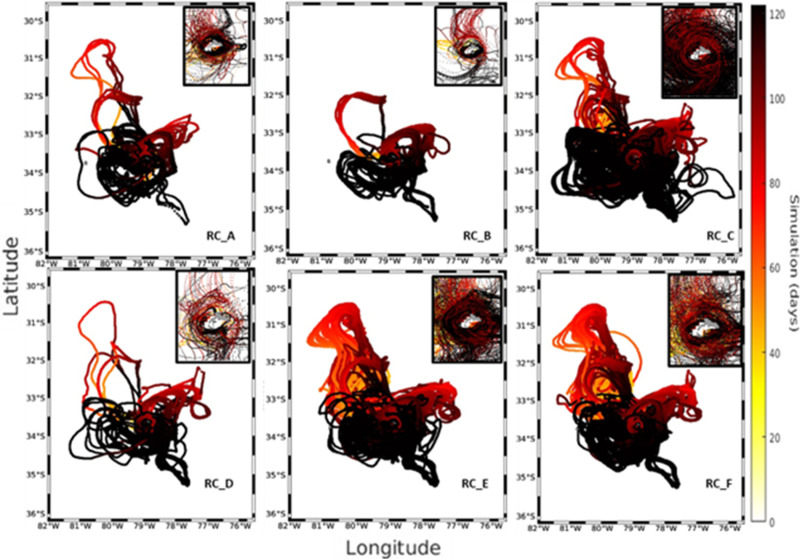
Trajectories of larvae arriving at the RC-SC recruitment zones for the year 2013. Each panel corresponds to the 6 zones used for the predictions, and the small plots show a close-up of the island to more clearly observe the movement patterns followed by the larvae. The color bar represents the days of the IBM simulation.

**Fig 4:**
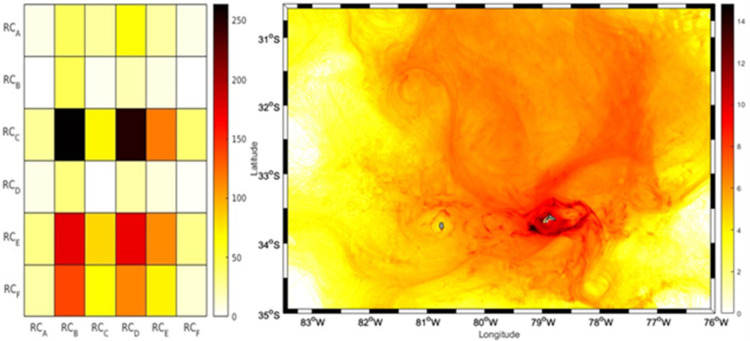
Connectivity matrix and larvae concentration for the year 2013. The left panel shows the connectivity matrix of IBM simulation experiments of *C. sylviae* larvae for 2013. On the x-axis the 6 release zones in RC are represented, on the y-axis the recruitment zones for RC are represented (6 zones). The colors represent the concentration of larvae at each site of interaction of the release zones with the recruitment zones. In the left panel of the figure a particle density map is displayed, showing in darker colors the sites with higher larval recurrences, The color bar represents the number of larvae.

In 2013, the trajectory of the larvae simulated during the first half of their larval stage (<60 days) exhibited a predominant advection towards the northeast region and northwest of the RC-SC islands (RC_A, RC_B, and RC_C), consistent with the mean current (S1 and S2 Figs). A circulation was observed primarily toward the southwest and southeast regions of the RC-SC islands (RC_D, RC_E, and RC_F) during the period of the second half of their larval stage, close to the recruitment period (>60 days). This circulation aided in larval retention and established connections with the same areas where the *C. sylviae* larvae were first released. The dispersive nature of the surrounding current system, notwithstanding, the successful particle trajectories to all zones indicated a distinct pattern of anticyclonic recirculation surrounding the islands. In comparisons of the three years, 2013 had the fewest recruits overall, with a total retention of 2120 larvae (Fig 3). The source region with the largest contribution (32.97%, or 699 larvae) in terms of the number of larvae recruited was the RC_B region, whereas the RC_C zone was the sink zone with the highest proportion (768 larvae or 36%). Regarding the larval accumulation surrounding RC-SC, the year 2013 exhibited particle presence along the island’s perimeter, with the northern and northwest portion (in general) of the island having the highest particle number (Fig 4).

**Fig 5:**
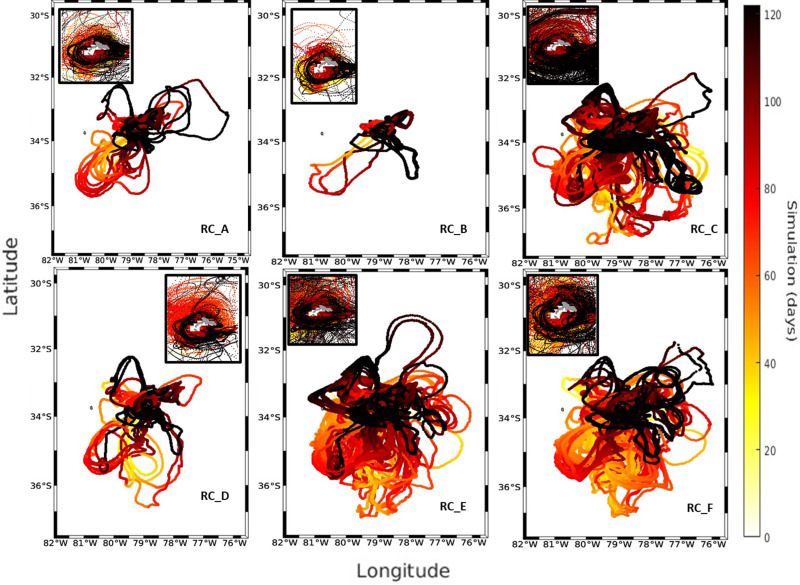
Trajectories of larvae arriving at the RC recruitment zones for the year 2015. Each panel is subdivided into each of the 6 zones used for the simulations. The small plots in each figure show a close-up of the island to more clearly observe the movement patterns followed by the larvae as they arrive at the recruitment zone. The color bar represents the days of the IBM simulation.

**Fig 6:**
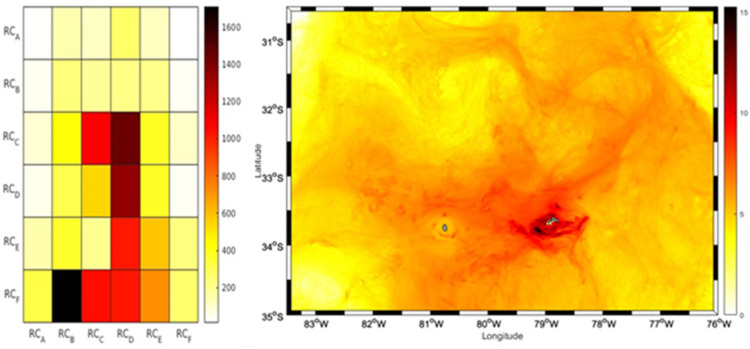
Connectivity matrix and larvae concentration for the year 2015. The right panel of the figure shows the connectivity matrix of IBM simulation experiments of *C. sylviae* larvae for 2015. On the x-axis the 6 release zones in RC are represented, on the y-axis the recruitment zones for RC are represented (6 zones). The colors represent the concentration of larvae at each site of interaction of the release zones with the recruitment zones. In the left panel of the figure a particle density map is displayed, showing in darker colors the sites with higher larval recurrences, The color bar represents the number of larvae.

In 2015, sub-mesoscale structures that promoted the advection of larvae off the coast were identified. The simulation of the first larval period exhibited predominant advection toward the southwest and southeast regions of the RC-SC islands (S3 and S4 Figs). Larval transit mostly took place in the northern zones second simulation period, exhibiting a link with the eastern sites (RC_E, RC_F) and western areas of the islands (RC_C; Fig 5). The RC_D zone had the highest percentage of contributed larvae (34.51%; 5316 larvae) among the total of released larvae (15403) identified in the time window as a recruitment proxy. However, the RC_F zone was a sink area, with the highest recruitment index reaching 32.95% (5076 larvae) of the larvae recruited. In contrast to 2013, the highest particle accumulation occurred in the eastern area of the islands (Fig 6).

**Fig 7:**
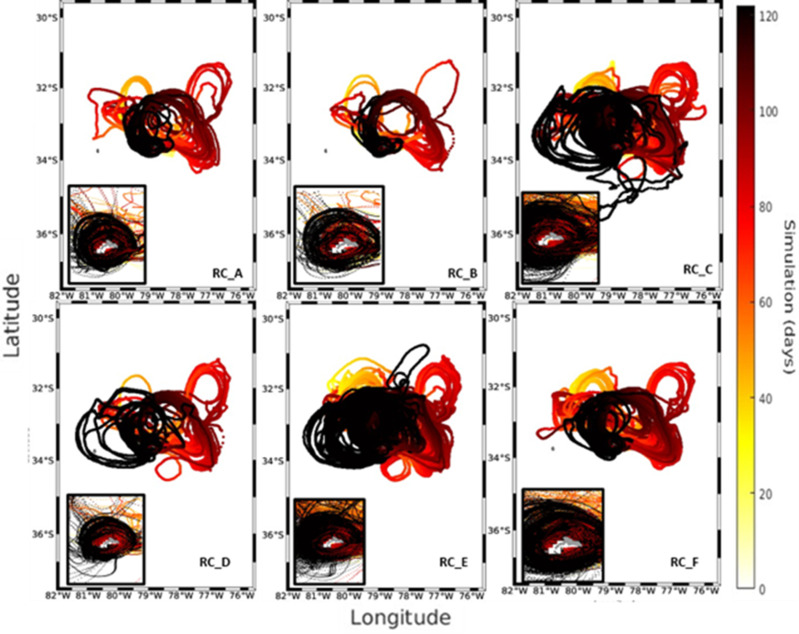
Trajectories of larvae arriving at the RC recruitment zones for the year 2018. Each panel is subdivided into each of the 6 zones used for the simulations. The small plots in each figure show a close-up of the island to more clearly observe the movement patterns followed by the larvae as they arrive at the recruitment zone. The color bar represents the days of the IBM simulation.

**Fig 8:**
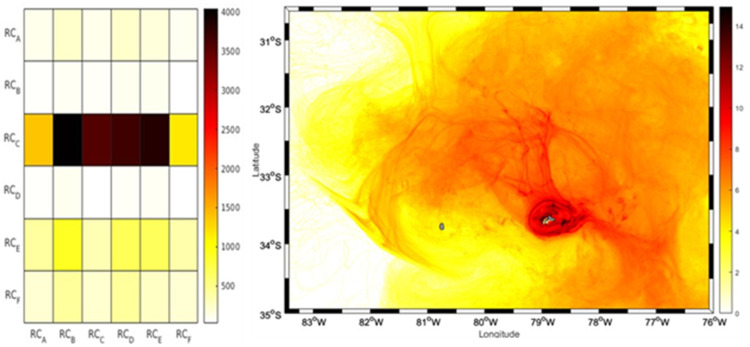
Connectivity matrix and larvae concentration for the year 2018. The right panel of the figure shows the connectivity matrix of IBM simulation experiments of *C. sylviae* larvae for 2018. On the x-axis the 6 release zones in RC are represented, on the y-axis the recruitment zones for RC are represented (6 zones). The colors represent the concentration of larvae at each site of interaction of the release zones with the recruitment zones. In the left panel of the figure a particle density map is displayed, showing in darker colors the sites with higher larval recurrences, The color bar represents the quantity of larvae.

Despite the predominance of the anticyclonic circulation pattern around the RC-SC islands in all years (S5 and S6 Figs), the extension of the transport of larvae out of the coast was limited in 2018 compared to the simulation cases for the years 2013 and 2015 (Fig 7). In this instance, the larval transport’s first phase (i.e., the first 60 days of larval dispersion) was primarily centered on the island’s northern regions; more than in the other three years. Regarding the six areas around the RC-SC islands, the RC_B zone had the highest contribution of recruited larvae (24%, or 5810 larvae), whereas the RC_C zone had the highest number of recruits (72.38%, or 17,676 larvae). As in 2013, most of the simulated larvae accumulated around the northern zones of the islands (Fig 8).

## Discussion

The results suggest that the demographic increase of *C. sylviae* in the JFA has been gradual and sustained over the last decade, particularly highlighting the period from 2018 onwards that experienced a considerable increase in the number of individuals present as bycatch from the *J. frontalis* fishery. Although individuals are present around the RC-CS island, the results of the biophysical experiments delineated areas that had greater concentrations of particles at the time of recruitment, areas that could potentially be sink zones for this species. The GLM results suggested a slight positive relationship between the presence of urchins present as bycatch and small size of lobsters, consistent with the *C. sylviae* and *J. frontalis* habitat distributions. The predator-prey relationship between species of sea urchins and lobsters has been previously documented [32], and thus these results may shed light on an interaction that requires further exploration.

### Spatial and temporal patterns

The distribution of adults of *C. sylviae* around RC-SC exhibited patterns that seemed to be strongly modulated by region-specific environmental and oceanographic factors. The analyses carried out from the fishery biological monitoring program of the lobster fisheries and the ecosystem associated with the JFA (2015–2022, [10]) indicate that the relative abundance of adult urchins shows two differentiated periods, one of low relative abundance (2015–2017) and a period where the population increased (2018–2022).

A strong El Niño event registered in the year 2015–2016 caused an increase of ~2°C in the equatorial region of the South Pacific system. Around the RC-SC island the average increase in sea surface temperature reached ~1°C in the austral summer, thus, adult individuals (and consequently their reproductive products), were exposed to positive temperature anomalies that may have contributed to an earlier release of gametes to the environment, potentially contributing to the to the demographic expansion of *C. sylviae* during year 2018. These climatic fluctuations modulate several factors (i.e., temperature, primary production, advection, and transport) that could affect the recruitment and subsequent distribution of adult urchins [33,34]. Currently, there are no records that document the effect of temperature on the distribution of *C. sylviae* adults. However, Hart and Scheibling [35] reported a decrease in the period of larval development of *S. droebachiensis* urchins at higher temperatures (within tolerance limits). This suggests that the increase in temperature in the water column could potentially have several effects on *C. sylviae*: 1) reduction of the larval period; 2) advancement of adulthood, and 3) advancement of sexual maturity. The above factors may also have influenced the larvae of *J. frontalis* in the archipelago [34]. Another relevant aspect to consider is that the relative abundance of urchins differs between the areas of the archipelago. This could be related to the presence of preferred microhabitats and more favorable conditions for their development and recruitment. In the northeast and northwest regions of RC-SC, more than 60% of the reefs are located on the island shelf, and most are distributed at depths between 0–150 m a factor that could provide refugia to a large marine community that includes *C. sylviae* [36]. In addition, it has been reported that *C. rodgersii*, a species related to *C. sylviae*, exhibits a preference for irregular substrates that provide shelter, a characteristic that also occurs in species that use reefs as niches [37]. The above coincides with the location of the RC_A, and RC_F zones (located in the north and northwest regions of RC-SC, respectively) that exhibited the highest relative abundance of urchins throughout the monitoring period.

The results of the analysis of the relative abundance of adult urchins were consistent with several aspects of the simulations of the dispersion and larval connectivity of the biophysical model for *C. sylviae*. In general, the simulation experiments exhibited significant interannual variability among the three years of study characterized by a high degree of dispersion and advection of the larvae off the coast of RC-SC. The RC_A zone had the greatest relative abundance of adult urchins, and according to the connectivity experiments, this zone retains a low number of particles. By the other hand, RC_B stands out for having the largest contribution of larvae to the other areas of RC-SC. Therefore, the RC_A and RC_B zone can plausibly be considered as sources, but not as a sink since they have minor value as a source of larval recruitment. The RC_E zone is one of three zones with the greatest relative abundance of adults, and this same zone was coincidentally the zone with the greatest particle retention in the simulation experiments for the years 2013 and 2018. Variation in the degree of larval recruitment can be modulated both by oceanographic aspects and characteristics of the early cycle of the species (duration of the planktonic phase, tolerance limits, or nutritional requirements). However, on average, all the areas of the island evaluated in this study are connected and thus support the hypothesis that the resident populations of *C. sylviae* constitute a single genetic population [16]. The mechanisms of retention and dispersal of planktonic organisms in the water column are important for understanding the genetics and connectivity of benthic populations in geographically isolated systems [38]. Even though for species of echinoderms the patterns of vertical migration or swimming [17] have been reported, there are no research data for *C. sylviae*. Thus, future modeling studies should consider other fundamental aspects such as the shape of the pluteus larvae since the extensions of the arms give it buoyancy as a mechanism to remain at shallower depths. Also, the capacity and swimming dynamics of the larvae, the physiological tolerance limits of both the adults and larvae, and other relevant factors determine not only the distribution patterns of the species but also the rates of mortality. Regarding the hydrodynamic models employed in the present study, it is recommended that future work consider the development of hydrodynamic models with higher spatial resolution to improve the resolution of the islands of the archipelago, the sub-mesoscale structures, and the local circulation patterns.

### Environmental relationship

Another aspect to consider concerning the colonization of the sea urchins in the RC-SC is the impact of the 2010 tsunami. This event generated an uprising of the seabed, causing an increase in the concentration of dissolved organic matter in the water column in addition to the removal of a considerable proportion of the fauna of the rocky intertidal zone, thus freeing up space for the potential recruitment of new individuals [39]. This could have facilitated the colonization of the subtidal substrates of algae species such as *Ulva* spp. This change in habitat could have provided a new food resource for the urchins, hence the demographic increase and the subsequent contribution to the progressive whitening of the island sea bottoms [40]. In contrast, the decrease in starfish by generations of fishing could have also contributed to decreasing the control of the sea urchin population (J. Chamorro, personal communication). Even though large lobsters could play a role similar to that of starfish, the commercial exploitation of *J. frontalis* has resulted in the extraction of a high percentage of lobsters over the allowed size, thereby decreasing potential biological control of the urchin population. Together, these elements outline a dynamic and complex panorama that may have contributed to the expansion of *C. sylviae* in the ecosystem [41].

### Ecological factors

A crucial factor to consider is the possible interaction between adult urchins and Juan Fernandez’s lobster. The coexistence of these two species raises questions concerning their potential ecological relationships (i.e., predation, resource competition), or the impact they may have on the community structure of the archipelago [42,43]. Changes in the minimum and maximum size restrictions for commercial lobster fishing could potentially contribute to population control of juvenile and small adult sea urchins in the study area. Specifically, these restrictions may allow for an increase in the biomass of large adult lobsters, which could exert predation pressure in these smaller size classes of *C. sylviae*. [44]. However, an increase in natural predators could result in a migration (both in depth and latitudinally) of adults to avoid predation in the long term [45]. It is also important to note that although there are documented cases of predation of sea urchins by lobsters (i.e., *Jasus edwardsii* and *Centrostephanus rodgersii*) in Tasmania [46], this does not imply that lobsters prefer urchins over other species. Lobsters that are distributed in sea urchin territory exhibit a greater consumption of urchins, but not at a level where they can control the growth of the urchin population [32], although this has not been studied in communities with a high degree of isolation as in the case of *J. frontalis* and *C. sylviae* in JFA. There are reports of decreased populations of sea urchins (*Strongylocentrotus droebachiensis*) due to the presence of starfish (*Pycnopodia*) and lobsters (Homarus americanus) [47], and thus these species could be key to the community structure present in the JFA. This suggests the need to evaluate management strategies based on ecosystem approaches using, for example, Intermediate complexity models [48], end-to-end models such as ecopath with ecosim [49] or Atlantis [11]. This will allow evaluation of the impact on the ecosystem level of strategies such as the removal of the urchins or the implementation of a maximum catch size of lobsters [10].

### Toward management and future work

This study highlights the importance of the biological interaction between *J. frontalis* and *C. sylviae*, which might have a large impact on the benthic marine ecosystem of Juan Fernández. Sea urchin abundance outbreaks have produced large barrens in rocky reef ecosystems in Juan Fernández and elsewhere, therefore there is a need for comprehensive laboratory and field experiments to assess predation of sea urchins by lobsters, especially to characterize the size-specific predator/prey relationship. For instance, genetic analyses of lobster feces [50] and stable isotopes of other body tissues could provide insights into the presence of sea urchin DNA and other prey, shedding light on these complex ecological interactions.

Improving the ecological information on functional group interaction in the Juan Fernández vulnerable ecosystem [51] is a priority. The end-to-end ecosystem modelling framework [52] is the best available scientific tool for evaluating human impacts at an ecosystem level. This may provide managers with proper strategic tools for defining adequate management actions in the emblematic Juan Fernandez lobster fishery, anticipating potential ecosystem shifts that may ultimately affect lobster productivity.

In recent years, the Fishing Council of the Main Artisanal Fisheries of Juan Fernández has decided to incorporate a maximum size for retained female lobsters of 140 mm of carapace length. Although this measure is geared toward increasing the stock’s reproductive potential, it will probably also benefit the predatory interaction between larger females and sea urchins. As more predatory ecological data becomes available, new regulations on maximum size restrictions for males may be proposed.

Despite these advances, the lack of higher spatiotemporal resolution models for the study period represents a notable limitation. For one hand, there is a need for development of high resolution interannual variability models, and for the other to develop finer temporal resolution to capture tidal signals. Addressing these gaps in future research would enable a more detailed assessment of the impact of sub-mesoscale structures on larval dynamics within the JFA system. Nevertheless, the models employed in this study represent the best available product to date, offering daily temporal resolution that adequately captures the hydrodynamic context in remote areas like the JFA region.

## Supporting information

S1 Fig Zonal velocities (positive: east; negative: west) for the larval distribution period of *C. sylviae* for the year 2013. (AMJ (autumn): April, May, and June; JAS (winter): July, August, and September; OND (spring): October, November, and December), averaged up to 100 (m) considering the study area (JFA-ID). Data obtained from the Copernicus model.
(TIF)

S2 Fig Meridional velocities: (positive: north; negative: south) for the larval distribution period of *C. sylviae* for the year 2013. (AMJ (autumn): April, May, and June; JAS (winter): July, August, and September; OND (spring): October, November, and December), averaged up to 100 (m) considering the study area (JFA-ID). Data obtained from the Copernicus model.
(TIF)

S3 Fig Zonal velocities (positive: east; negative: west) for the larval distribution period of *C. sylviae* for the year 2015. (AMJ (autumn): April, May, and June; JAS (winter): July, August, and September; OND (spring): October, November, and December), averaged up to 100 (m) considering the study area (JFA-ID). Data obtained from the Copernicus model.
(TIF)

S4 Fig Meridional velocities: (positive: north; negative: south) for the larval distribution period of *C. sylviae* for the year 2015. (AMJ (autumn): April, May, and June; JAS (winter): July, August, and September; OND (spring): October, November, and December), averaged up to 100 (m) considering the study area (JFA-ID). Data obtained from the Copernicus model.
(TIF)

S5 Fig Zonal velocities (positive: east; negative: west) for the larval distribution period of *C. sylviae* for the year 2018. (AMJ (autumn): April, May, and June; JAS (winter): July, August, and September; OND (spring): October, November, and December), averaged up to 100 (m) considering the study area (JFA-ID). Data obtained from the Copernicus model.
(TIF)

S6 Fig Meridional velocities: (positive: north; negative: south) for the larval distribution period of *C. sylviae* for the year 2018. (AMJ (autumn): April, May, and June; JAS (winter): July, August, and September; OND (spring): October, November, and December), averaged up to 100 (m) considering the study area (JFA-ID). Data obtained from the Copernicus model.
(TIF)

S7 Fig Data based on the monitoring program showing the presence of *C. sylviae* as bycatch per lobster trap around RC-SC. The upper panel shows the presence of sea urchins of the year 2015 (beginning of the monitoring program). The lower panel shows the presence of sea urchins of the year 2022 (the end of the time series of the monitoring program). Figure generated under a CC BY license, with permission from Billy Ernst, original copyright 2024.
(TIF)

S8 Fig Superficial temperature anomalies of the year 2015 around RC-SC system of the of the hydrodynamic model coupled to IBM experiments. (TIF)

S9 Fig Model grid and mask of the hydrodynamic model coupled with IBM experiments used to represent the circulation of the study area (RC-SC in JFA). (TIF)

S1 Table Relative abundance of sea urchin GLM results from all zones separately. Results of models fit a relative abundance response variable (sea urchin) using simple (*m*) and multiple (*M*) regression models by zone, where the covariables are year factor (*a*_*j*_), month (*m*_*k*_) depth (*d*_*m*_) and rock lobster size (*s*_*n*_). AIC corresponds to Akaike information criteria and df to degrees of freedom. Bold and italic indicate best model fit for each level.
(DOCX)

S2 Table Summary table of generalized linear model results of the best fit model (M5). Results of the best model fit a relative abundance response variable (sea urchin) using multiple (*M*) regression models, where the covariables are year factor (*a*_*j*_), month (*m*_*k*_) depth (*d*_*m*_) and rock lobster size (*s*_*n*_). Bold and italic indicate the value of the estimated coefficients.
(DOCX)
