# Supplementary figures and images for: Demographic rise of sea urchin Centrostephanus sylviae on Robinson Crusoe and Santa Clara Islands at the Juan Fernandez Archipelago: A biophysical and ecological approach
Source: PLoS One. 2025 Jun 25;20(6):e0325556. doi: 10.1371/journal.pone.0325556 (PMC12194239; doi:10.1371/journal.pone.0325556)

**
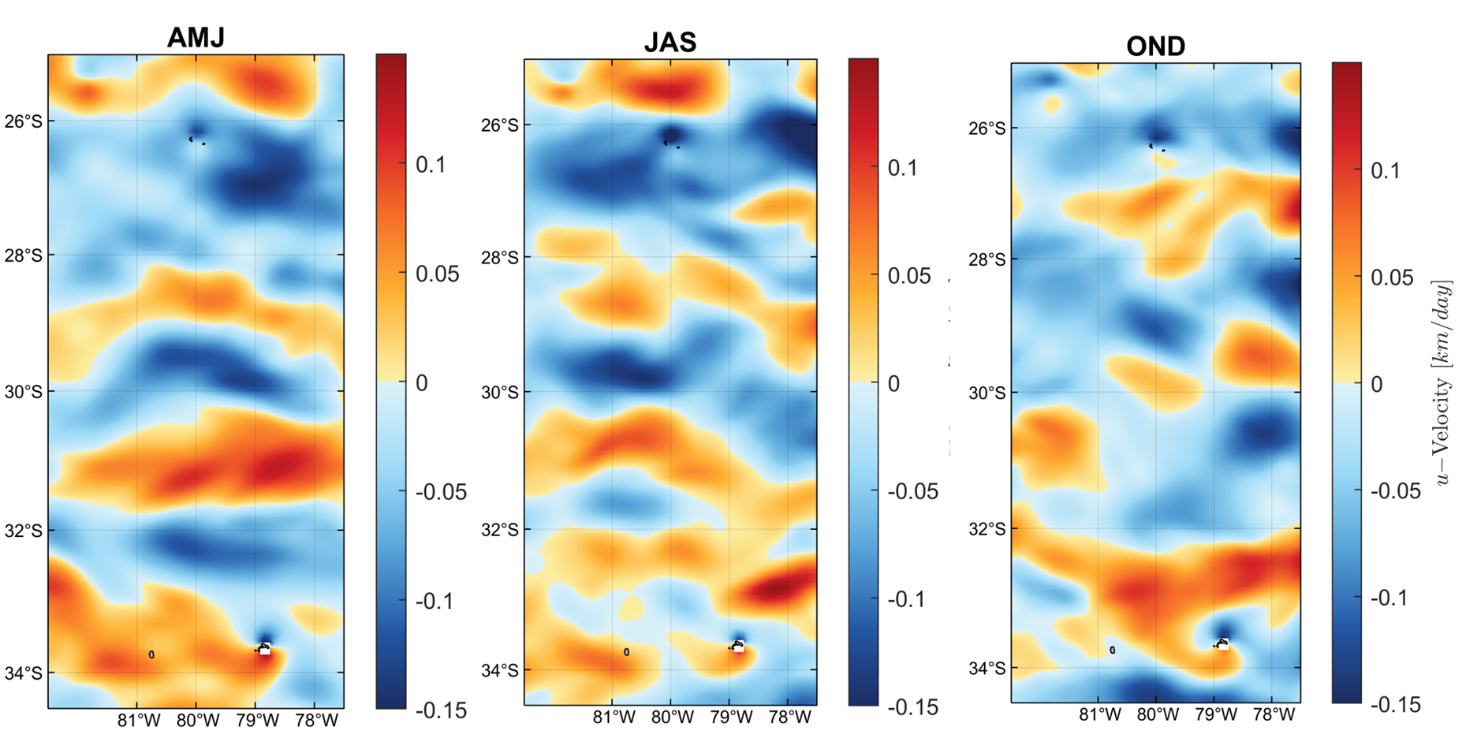
**

Supplement: S1 Fig — (AMJ (autumn): April, May, and June; JAS (winter): July, August, and September; OND (spring): October, November, and December), averaged up to 100 (m) considering the study area (JFA-ID). Data obtained from the Copernicus model. (TIF) [file pone.0325556.s001.docx]

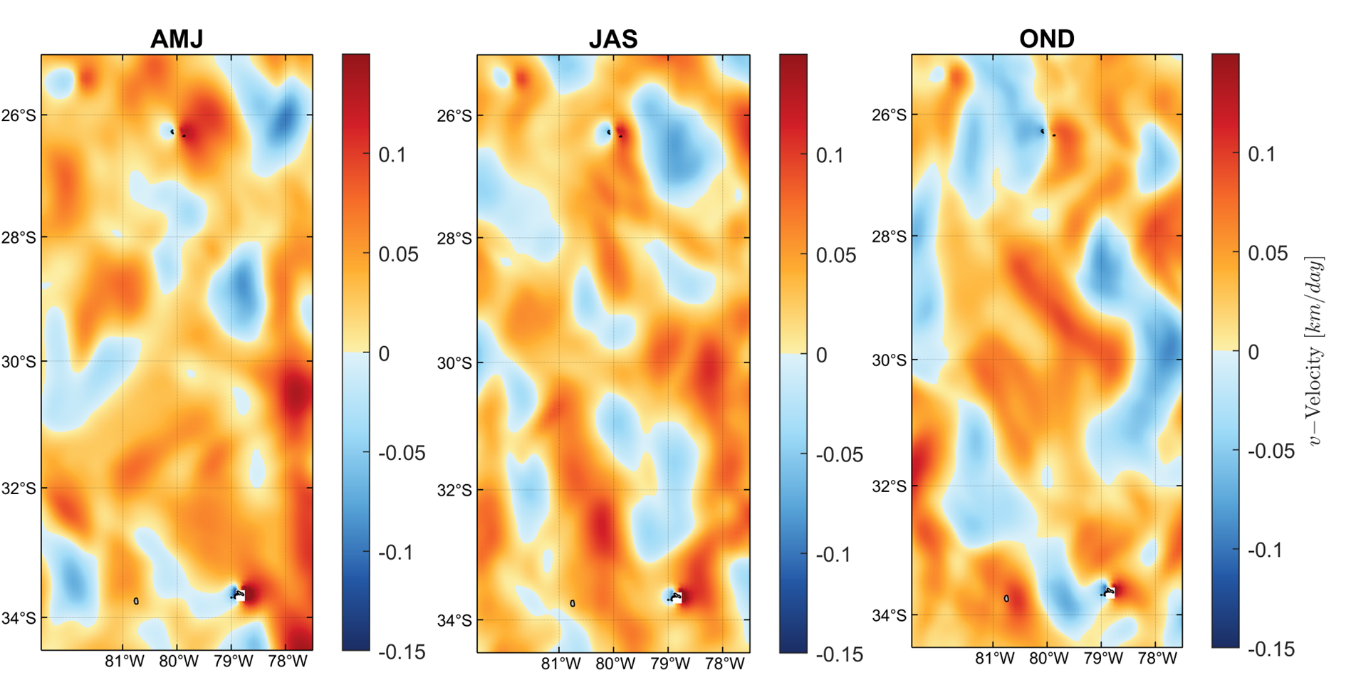

Supplement: S2 Fig — (AMJ (autumn): April, May, and June; JAS (winter): July, August, and September; OND (spring): October, November, and December), averaged up to 100 (m) considering the study area (JFA-ID). Data obtained from the Copernicus model. (TIF) [file pone.0325556.s002.docx]

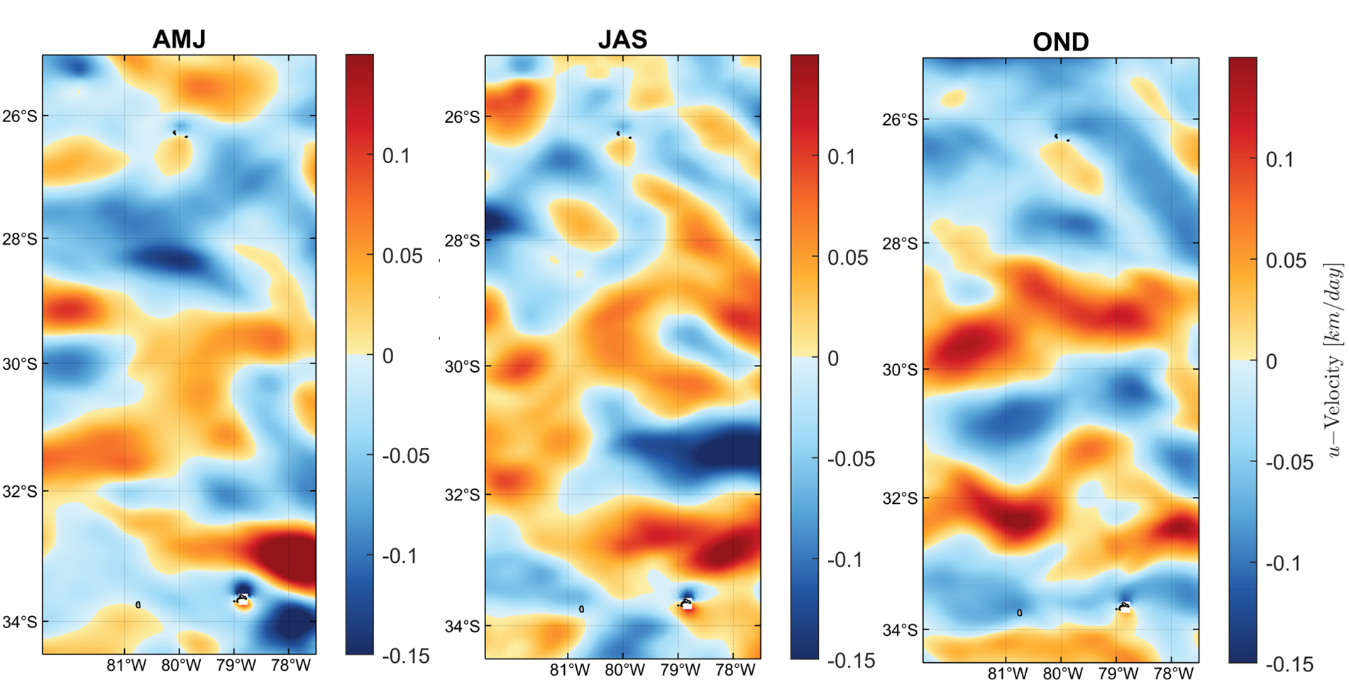

Supplement: S3 Fig — (AMJ (autumn): April, May, and June; JAS (winter): July, August, and September; OND (spring): October, November, and December), averaged up to 100 (m) considering the study area (JFA-ID). Data obtained from the Copernicus model. (TIF) [file pone.0325556.s003.docx]

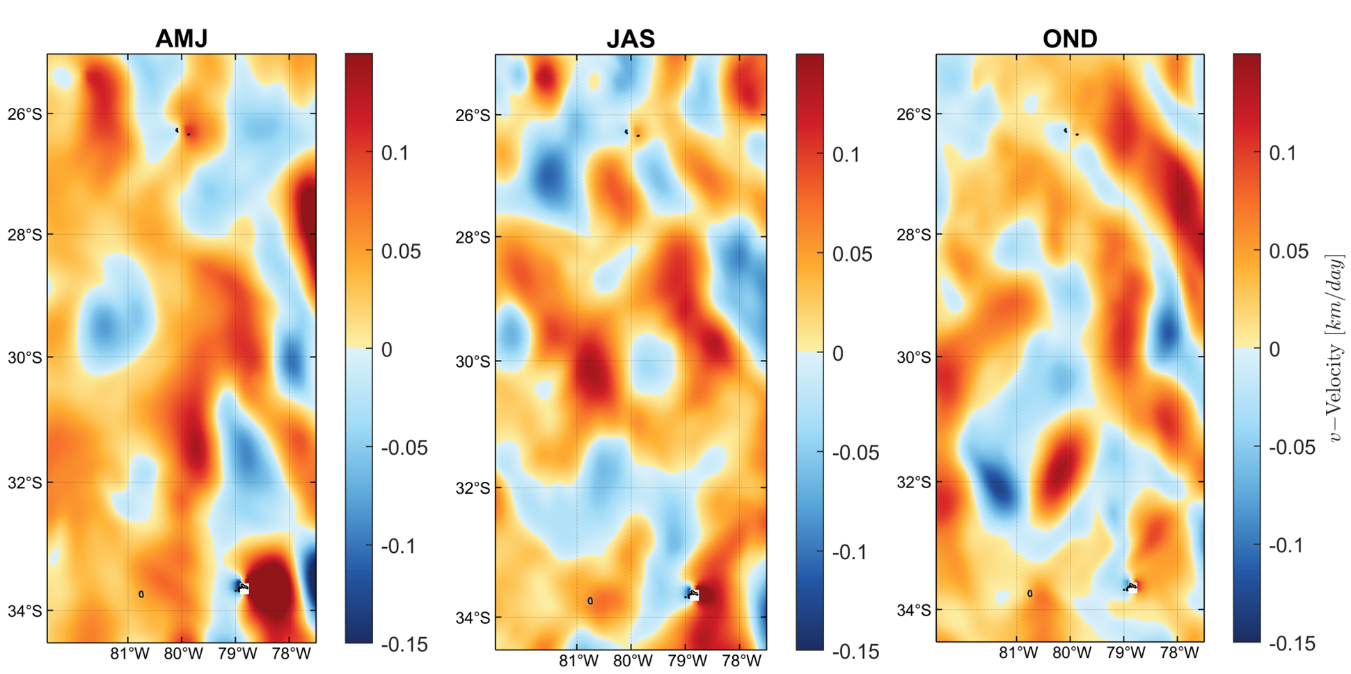

Supplement: S4 Fig — (AMJ (autumn): April, May, and June; JAS (winter): July, August, and September; OND (spring): October, November, and December), averaged up to 100 (m) considering the study area (JFA-ID). Data obtained from the Copernicus model. (TIF) [file pone.0325556.s004.docx]

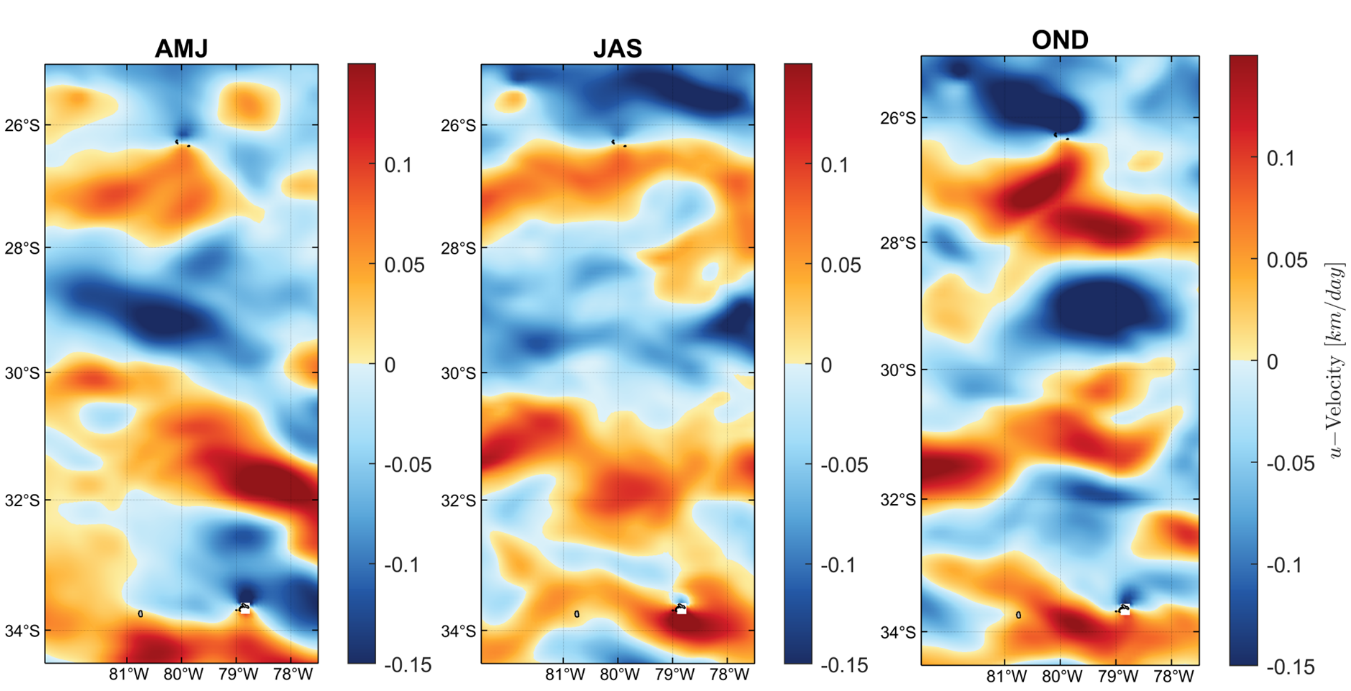

Supplement: S5 Fig — (AMJ (autumn): April, May, and June; JAS (winter): July, August, and September; OND (spring): October, November, and December), averaged up to 100 (m) considering the study area (JFA-ID). Data obtained from the Copernicus model. (TIF) [file pone.0325556.s005.docx]

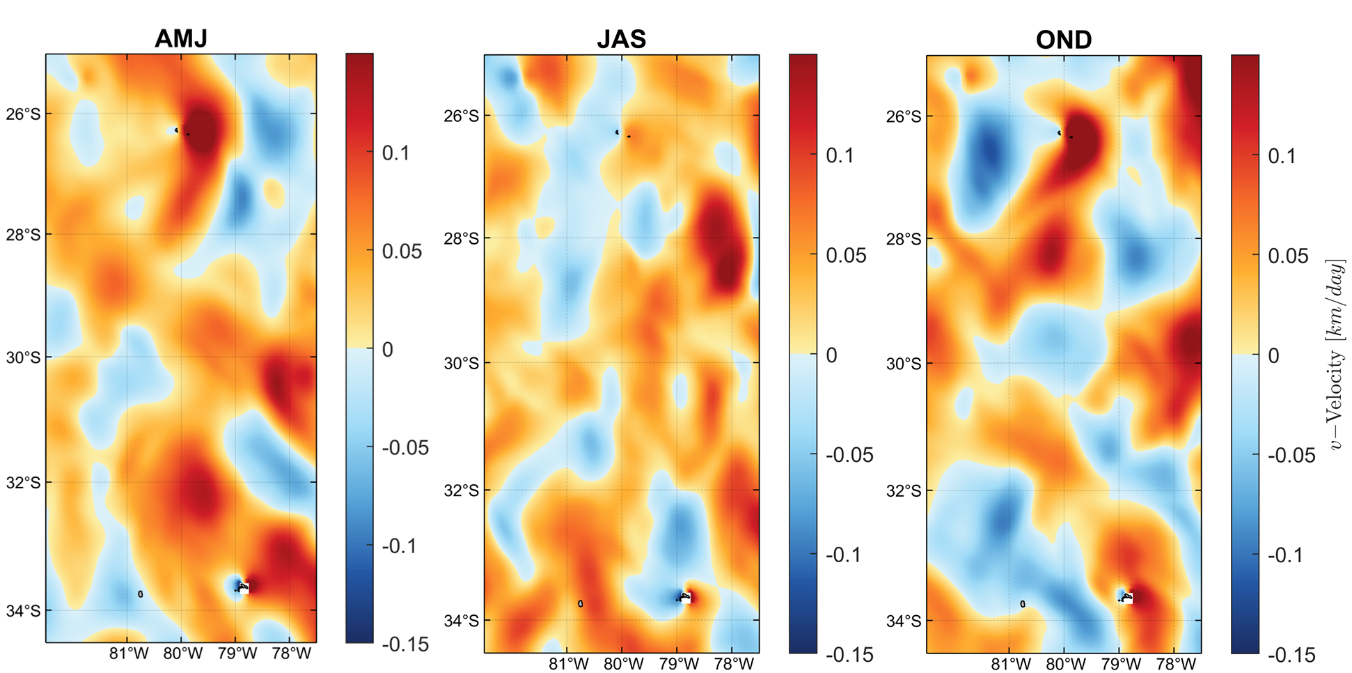

Supplement: S6 Fig — (AMJ (autumn): April, May, and June; JAS (winter): July, August, and September; OND (spring): October, November, and December), averaged up to 100 (m) considering the study area (JFA-ID). Data obtained from the Copernicus model. (TIF) [file pone.0325556.s006.docx]

**
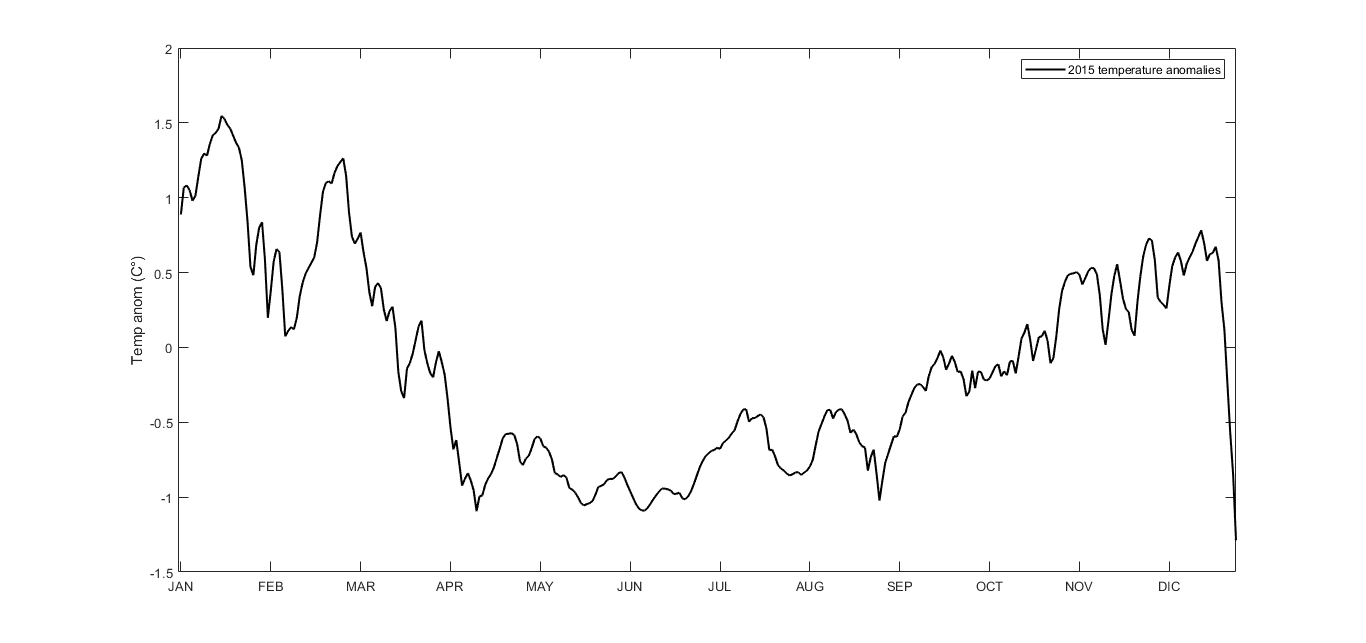
**

Supplement: S8 Fig — (TIF) [file pone.0325556.s008.docx]

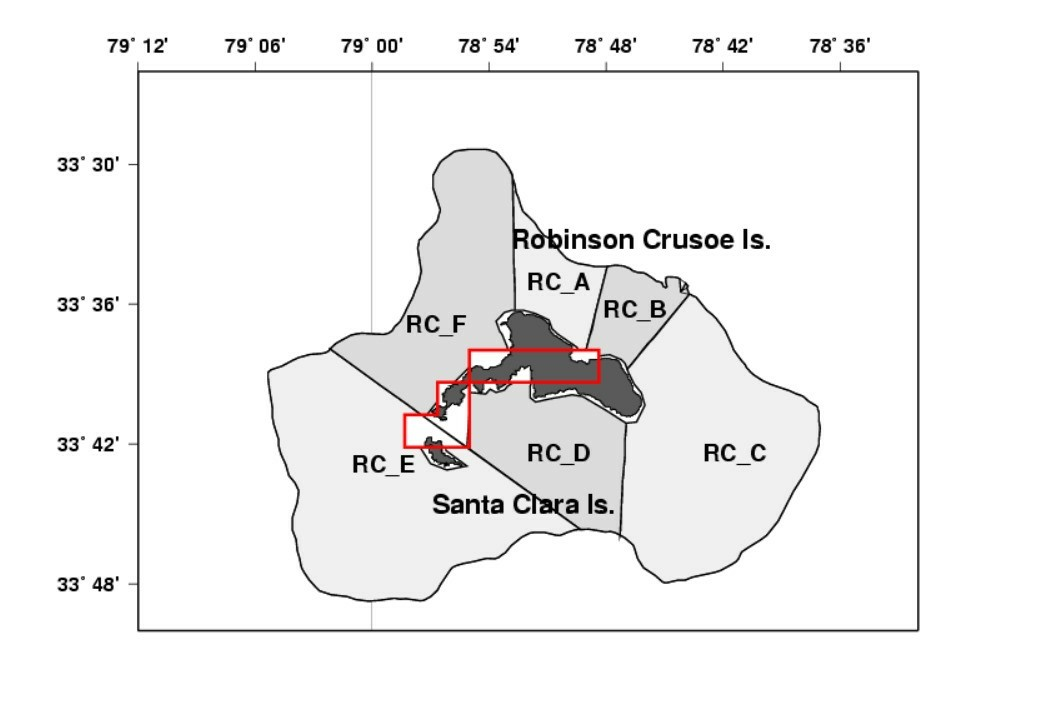

Supplement: S9 Fig — (TIF) [file pone.0325556.s009.docx]
