# Supplementary material for: Demographic rise of sea urchin Centrostephanus sylviae on Robinson Crusoe and Santa Clara Islands at the Juan Fernandez Archipelago: A biophysical and ecological approach
Source: PLoS One. 2025 Jun 25;20(6):e0325556. doi: 10.1371/journal.pone.0325556 (PMC12194239; doi:10.1371/journal.pone.0325556)
Supplement: S1 Table — Results of models fit a relative abundance response variable (sea urchin) using simple (m) and multiple (M) regression models by zone, where the covariables are year factor (aj), month (mk) depth (dm) and rock lobster size (sn). AIC corresponds to Akaike information criteria and df to degrees of freedom. Bold and italic indicate best model fit for each level. (DOCX) [file pone.0325556.s010.docx]

**S1 Table. Relative abundance of sea urchin GLM results from all zones separately**. Results of models fit a relative abundance response variable (sea urchin) using simple (*m*) and multiple (*M*) regression models by zone, where the covariables are year factor (*a_j_*), month (*m_k_*) depth (*d_m_*) and rock lobster size (*s_n_*). AIC corresponds to Akaike information criteria and df to degrees of freedom. Bold and italic indicate best model fit for each level.

| Simple regression | | | | | Multiple regression | | | |
| --- | --- | --- | --- | --- | --- | --- | --- | --- |
| **Zone** | **Model** | **Covariate** | **df** | **AIC** | **Model** | **Covariate** | **df** | **AIC** |
| RC_A | m0 | ~1 | 3 | 3960 | M0 | ~1 | 3 | 3960 |
|  | **m1** | **~year** | **10** | **3712** | M1 | **a_j_** | 10 | 3712 |
|  | m2 | ~month | 10 | 3881 | M2 | ~**a_j_** + m_k_ | 17 | 3616 |
|  | m3 | ~depth | 6 | 3881 | M3 | ~**a_j_** + m_k_ + d_m_ | 20 | 3602 |
|  | m4 | ~size | 4 | 3961 | **M4** | **~a_j_** + m_k_ + d_m_ **+** s_n_ | **21** | **3603** |
| RC_B | m0 | ~1 | 3 | 743 | M0 | ~1 | 3 | 743 |
|  | **m1** | **~year** | **10** | **687** | **M1** | ~**a_j_** | 10 | **687** |
|  | m2 | ~month | 10 | 746 | M2 | ~**a_j_** + m_k_ | 17 | 690 |
|  | m3 | ~depth | 6 | 748 | M3 | ~**a_j_** + m_k_ + d_m_ | 20 | 695 |
|  | m4 | ~size | 4 | 744 | M4 | **~a_j_** + m_k_ + d_m_ **+** s_n_ | **21** | 690 |
| RC_C | m0 | ~1 | 3 | 1122 | M0 | ~1 | 3 | 1122 |
|  | **m1** | **~year** | **10** | **1026** | M1 | ~**a_j_** | 10 | 1026 |
|  | m2 | ~month | 10 | 1093 | M2 | ~**a_j_** + m_k_ | 17 | 996 |
|  | m3 | ~depth | 6 | 1063 | M3 | ~**a_j_** + m_k_ + d_m_ | 20 | 973 |
|  | m4 | ~size | 4 | 1123 | **M4** | **~a_j_** + m_k_ + d_m_  **+** s_n_ | **21** | **974** |
| RC_D | m0 | ~1 | 3 | 1998 | M0 | ~1 | 3 | 1998 |
|  | **m1** | **~year** | **10** | **1881** | M1 | ~**a_j_** | 10 | 1881 |
|  | m2 | ~month | 10 | 1965 | M2 | ~**a_j_** + m_k_ | 17 | 1856 |
|  | m3 | ~depth | 6 | 1964 | M3 | ~**a_j_** + m_k_ + d_m_ | 20 | 1842 |
|  | m4 | ~size | 4 | 1997 | **M4** | **~a_j_** + m_k_ + d_m_ **+** s_n_ | **21** | **1842** |
| RC_E | m0 | ~1 | 3 | 3203 | M0 | ~1 | 3 | 3203 |
|  | **m1** | **~year** | **10** | **2968** | M1 | ~**a_j_** | 10 | 2968 |
|  | m2 | ~month | 10 | 3181 | M2 | ~**a_j_** + m_k_ | 17 | 2951 |
|  | m3 | ~depth | 6 | 3056 | M3 | ~**a_j_** + m_k_ + d_m_ | 20 | 2869 |
|  | m4 | ~size | 4 | 3193 | **M4** | **~a_j_** + m_k_ + d_m_ **+** s_n_ | **21** | **2865** |
| RC_F | m0 | ~1 | 3 | 2648 | M0 | ~1 | 3 | 2648 |
|  | **m1** | **~year** | **10** | **2437** | M1 | ~**a_j_** | 10 | 2437 |
|  | m2 | ~month | 10 | 2628 | M2 | ~**a_j_** + m_k_ | 17 | 2418 |
|  | m3 | ~depth | 6 | 2516 | M3 | ~**a_j_** + m_k_ + d_m_ | 20 | 2332 |
|  | m4 | ~size | 4 | 2639 | **M4** | **~a_j_** + m_k_ + d_m_ **+** s_n_ | **21** | **2332** |
