# Supplementary material for: Demographic rise of sea urchin Centrostephanus sylviae on Robinson Crusoe and Santa Clara Islands at the Juan Fernandez Archipelago: A biophysical and ecological approach
Source: PLoS One. 2025 Jun 25;20(6):e0325556. doi: 10.1371/journal.pone.0325556 (PMC12194239; doi:10.1371/journal.pone.0325556)
Supplement: S2 Table — Results of the best model fit a relative abundance response variable (sea urchin) using multiple (M) regression models, where the covariables are year factor (aj), month (mk) depth (dm) and rock lobster size (sn). Bold and italic indicate the value of the estimated coefficients. (DOCX) [file pone.0325556.s011.docx]

**S2 Table. Summary table of generalized linear model results of the best fit model (M5)**. Results of the best model fit a relative abundance response variable (sea urchin) using multiple (*M*) regression models, where the covariables are year factor (*a_j_*), month (*m_k_*) depth (*d_m_*) and rock lobster size (*s_n_*). Bold and italic indicate the value of the estimated coefficients.

|  | Estimate | Std. Error | z value | Pr(>\|z\|) |
| --- | --- | --- | --- | --- |
| (Intercept) | -2.749585 | 0.337866 | -8.138 | 4.02e-16 *** |
| Year2016 | -0.480882 | 0.435621 | -1.104 | 0.269636 |
| Year2017 | -0.307527 | 0.496053 | -0.620 | 0.535291 |
| Year2018 | 2.611183 | 0.300150 | 8.700 | < 2e-16 *** |
| Year2019 | 2.385065 | 0.296267 | 8.050 | 8.25e-16 *** |
| Year2020 | 2.977225 | 0.292650 | 10.173 | < 2e-16 *** |
| ***Year2021*** | ***3.439955*** | ***0.288816*** | ***11.911*** | ***< 2e-16 ****** |
| Year2022 | 3.124251 | 0.290086 | 10.770 | < 2e-16 *** |
| Month11 | -0.036285 | 0.123607 | -0.294 | 0.769099 |
| Month12 | 0.307601 | 0.117259 | 2.623 | 0.008709 ** |
| Month1 | 0.399968 | 0.121214 | 3.300 | 0.000968 *** |
| Month2 | 0.154941 | 0.141579 | 1.094 | 0.273790 |
| Month3 | -0.365606 | 0.167649 | -2.181 | 0.029199 * |
| Month4 | -0.542347 | 0.193492 | -2.803 | 0.005064 ** |
| Month5 | 0.086300 | 0.278837 | 0.310 | 0.756941 |
| RC_B | 0.291956 | 0.164416 | 1.776 | 0.075780 . |
| RC_C | -0.384474 | 0.145698 | -2.639 | 0.008319 ** |
| RC_D | -1.193924 | 0.114742 | -10.405 | < 2e-16 *** |
| RC_E | -0.441501 | 0.098009 | -4.505 | 6.65e-06 *** |
| RC_F | -0.928297 | 0.102084 | -9.093 | < 2e-16 *** |
| -100 (m) | 0.004437 | 0.083299 | 0.053 | < 2e-16 *** |
| -150 (m) | -2.178958 | 0.226464 | -9.622 | < 2e-16 *** |
| -200 (m) | -0.906151 | 0.602049 | -1.505 | 0.132295 |
| Size.small | 0.167127 | 0.070002 | 2.387 | 0.016965 * |

*p value*: 0 ‘***’ 0.001 ‘**’ 0.01 ‘*’ 0.05 ‘.’ 0.1 ‘ ’ 1
